# Microalgae-Driven Circular Agriculture: System Integration, Nutrient Recovery, and AI-Assisted Optimization

**DOI:** 10.3390/microorganisms14040753

**Published:** 2026-03-27

**Authors:** Xiaoyan Liu, Lijuan Wang, Chunyu Xing, Haiyan Liu, Guanghong Luo, Shenghui Yang

**Affiliations:** 1 Gansu Province Microalgae Technology Innovation Center, Hexi University, Zhangye 734000, China; hxwz2409@hxu.edu.cn (X.L.);; 2 Gansu Kaiyuan Biotechnology Development Center Co., Ltd., Hexi University, Zhangye 734000, China; 3 College of Agriculture and Ecological Engineering, Hexi University, Zhangye 734000, China; 4 College of Life Sciences and Engineering, Hexi University, Zhangye 734000, China

**Keywords:** microalgae, high-value products, integral biorefinery, Al-assisted modelling

## Abstract

With rising global pressures on resources and the environment, transitioning out of our traditional linear agricultural models is long overdue. By itself, circular agriculture seeks to close loops for nutrients, but it also has a future that is constrained by the fragmentation of process integration, lack of system integration and optimization, and poor adaptive decision-making under the often very variable circumstances of agricultural systems. Microalgae are a versatile photosynthetic platform with unique value in this context. They can recover key nutrients (nitrogen, phosphorus and carbon) from agricultural wastes simultaneously and also convert these vital nutrients into multipurpose biomass. Here, this review synthesizes the multifunction of microalgae towards sustainable agriculture, with a particular emphasis on nutrient recycling and the use of whole microalgal biomass. Downstream applications are manifold, ranging from agricultural outputs, such as biofertilizers and biostimulants, to different products of high value (HVPs). Realizing this potential requires practical challenges to be addressed in integrated system design, coupling and scaling up. AI-assisted modelling and optimization have already started emerging as important tools for this purpose. Reliable system optimization relies on defining objective functions and balancing resource recovery efficiency and economic output, which in turn enables robust multi-objective decision-making. Concluding this review, we propose a holistic vision from a central integral biorefinery concept. Our framework clearly demonstrates how to fully enhance competitiveness, sustainability and scalability of microalgae-based agricultural systems through co-integrated high-value utilization and nutrient cycling.

## 1. Introduction

In the global agricultural production system, the long-dominant linear growth model is characterized by high-intensity external inputs and a heavy reliance on chemical fertilizers to sustain crop productivity [1]. While this model has substantially increased food supply capacity, it has simultaneously driven the accumulation of resource inefficiencies and environmental externalities [2]. These challenges are manifested in accelerated losses of soil nitrogen and phosphorus, the accumulation of agricultural wastes, heightened risks of aquatic eutrophication [3], and a sustained increase in greenhouse gas emissions [4]. As pressure on arable land, water resources and key mineral nutrients (in particular, phosphorus) becomes greater [5], the inefficiency of linear agriculture for resource use, environmental and productivity sustainability and medium- to long-term stability is becoming increasingly apparent. Clearly, the sustainable conversion of agriculture is not just a straightforward replacement of input factors; it is a big step of optimization on material flow, energy efficiency and ecological carrying capacity. Therefore, due to intensified resource constraint conditions and the continuous aggravation of agricultural non-point pollution pressures, a transformation toward a production mode that effectively consumes resources and holds low carbon emissions is imminent. Circular agriculture is the main path of such a transformation. It aims to break the constraints of linear production modes by building a circular system for the effective utilization of nutrients and resources. However, at present, circular agriculture technologies emphasize only the optimization of various links and generally do not involve cross-scale linkage of the system and coordinated control of multiple objectives.

The circular economy provides a robust conceptual basis to redesign agricultural systems by highlighting closed material and energy flows, waste valorization and nutrient recycling [6]. In the circular economy framework, circular agriculture represents steps towards decoupling from finite inputs by the resource-oriented transformation of agricultural wastes and recurrence of recovered nutrients into agricultural production processes [3,7]. In principle, such approaches can evolve from linear farming systems to integrated bioprocessing systems, in which waste regeneration, resource recycling and cascade usage occur closely coupled [8,9]. However, in practice, many existing circular agriculture technologies are still fragmented and are technology- and function-based, addressing isolated technologies such as composting [10], anaerobic digestion for biogas production, or wastewater treatment [11]. While each of them can yield some degree of nutrient recovery (up to 80% in the case of anaerobic digestion [12]), they often entail additional energy costs and often lack effective integration across multiple resource flows [13]. Hence, their overall system performance and potential value amplification are limited, which restricts the full synergistic deployment of environmental and economic advantages. As a result, these technologies do not yet benefit from integrated techno-economic objective functions and integrated cross-process coordination, and hence cannot operate as whole-system optimized solutions and scale-up.

Microalgae have attracted increasing interest in agriculture and environmental engineering because they are highly efficient photoautotrophic platforms for waste treatment and resource recovery [14,15]. Microalgal systems can use agricultural wastewater, animal farm wastewater and CO_2_-rich gas as growth substrates [16] and effectively assimilate inorganic N, P and C [17]. These products can be converted by the photosynthesis of microalgal cells to rich multipurpose biomass from proteins, lipids, polysaccharides and other bioactive substances [18], yielding products that have direct links to agriculture. Importantly, microalgae technologies have the opportunity to seamlessly combine waste recovery, nutrient management and the production of agrochemicals into a single operation platform [19]. Microalgae biomass and by-products can be further used as biofertilizers, biostimulants and soil ameliorants [20], which lead to improved soil quality, enhanced crop yields and raised crop stress resistance [21]. Microalgae deliver fixed carbon and nutrients back to cropland and close this loop as a “waste–microalgae–cropland” cycle [22], making them a hub function of circular agriculture [23,24]. Other than nutrient recovery, microalgae have significant metabolic plasticity, and thus, can be used to produce HVPs on demand, making microalgae a potential biological heart of an integrated biorefinery. Such a dual function—nutrient recovery and value exploitation—sets microalgae apart from common waste treatment technologies, making them more relevant for the circular agricultural transformation.

Despite the above benefits, existing studies on microalgae in circular agriculture concentrate on single application cases or studies of mechanism at the lab scale, but pay less attention to system integration, process coupling and comprehensive performance. Specific comparative evaluation among different microalgae application modes—in terms of nutrient recovery rate, energy demand and agro-synergy—is lacking. Furthermore, insufficient emphasis is given to the biological starting point of system design: the strain selection and genetic potential assessment, which define the nutrient conversion efficiency and product quality, as well as possible degrees of adaptation to the agricultural environment. As the microalgal system scales up and competes with a heterogeneous agricultural infrastructure, the complexity of operating factors markedly increases, which calls for more complex and advanced tools facilitating multivariate control and dynamic decision-making. Building on the above, AI-assisted modelling and multi-objective optimization are emerging not just as complementary tools but also as important building blocks to accomplish integrated “strain–process–system” coordination. Incorporating nutrient recovery rate, energy consumption, HVPs generation and economic income to a unified objective function, AI-driven optimization has the capability to conduct balanced optimization under environmental and resource constraints. Accordingly, this review portrays microalgae as a central integrative platform for circular agricultural systems and presents the systematic engineering role of microalgae.

## 2. Microalgae-Driven Resource Utilization and Nutrient Recovery from Agricultural Waste

### 2.1. Agricultural Waste as an Engineering Challenge for Nutrient Recovery

Green agricultural production systems create enormous volumes of nutrient-rich waste, such as livestock and poultry [25], effluent, agricultural runoff and processing process wastewater [26]. Technically, such waste streams for agriculture are different from ‘urban’ point source pollution in that they are spatially distributed, temporally intermittent and closely tied to the seasonality of agricultural activities [27]. All these factors are likely to contribute to very high collection and transport costs, fluctuations in pollutant loads and heterogeneous physicochemical characteristics, which together make centralized treatment and resource recovery a very hard problem [28].

Despite their dilute and variable nature, agricultural wastes contain significant amounts of recoverable nitrogen, phosphorus [29], and organic matter, indicating considerable theoretical potential for nutrient recycling and energy recovery [30]. However, the intrinsic features of “low concentration, large volume, and unstable composition” fundamentally constrain the performance of conventional treatment technologies [31]. Processes such as activated sludge systems and anaerobic digestion are primarily designed for regulatory compliance and pollutant removal, rather than for maximizing nutrient retention or downstream resource utilization. As a result, nutrients are frequently dissipated through gaseous emissions, immobilized in sludge, or unevenly redistributed, hindering the establishment of closed-loop nutrient cycles at the agricultural system level [32].

These limitations are further aggravated in practice by energy-intensive operational needs [33,34], such as continuous aeration and heat control, rendering technology economically unattractive in a decentralized rural setting [35]. As a result, there is emerging awareness that conventional, single-function treatment technologies are incapable of fulfilling the two tasks, large-scale agricultural waste treatment and efficient resource valorization. What is needed is an integrated [36], low energy-based technological concept that can simultaneously control pollutant discharge, recover nutrients and generate biomass even under highly varying operating conditions.

### 2.2. Process Mechanisms and Performance Advantages of Microalgae-Based Systems

Microalgae-based systems provide an entirely different engineering approach for agricultural waste management by linking wastewater treatment to biomatter production through photosynthetic metabolism [37]. Due to their highly efficient photosynthesis, microalgae can use inorganic carbon in water (CO_2_ and bicarbonate) and convert it into organic carbon required for growth [38]. At the same time, microalgae can quickly absorb and assimilate dissolved nutrient components, such as nitrate, ammonium and soluble phosphate [39], so that microalgae can effectively treat pollutants and realize nutrient extraction simultaneously [37,40]. This concept redefines agricultural wastes from a waste disposal issue into a biotechnological feedstock for biological synthesis. Nevertheless, interspecific differences among microalgal species or strains are substantial in terms of nutrient uptake efficacy and tolerance level, as well as metabolic pathways [41,42]. *Chlorella* [43,44,45], *Scenedesmus* [46,47] and *Spirulina* [48,49] are some widely investigated genera that show great adaption to enriched waste nutrients, and are thus used for the treatment of eutrophic waters like agricultural or aquaculture waste effluents [50].

**Figure 1:**
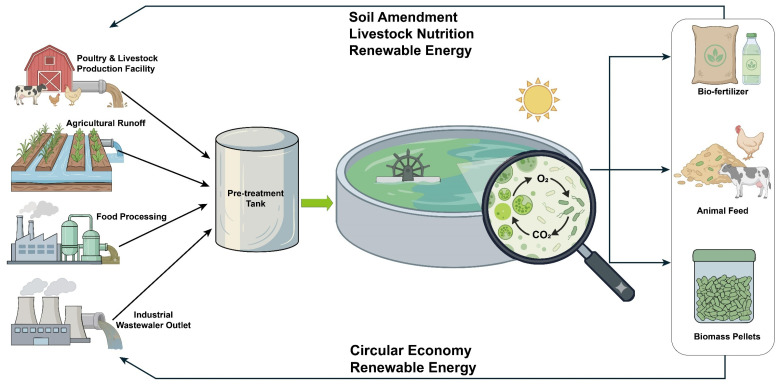
Schematic of a microalgae-based agricultural waste valorization and circular bioeconomy model (created by the authors based on the literature [18,55,56,57]).

Under optimal operating conditions, microalgae-based treatment systems achieve nutrient removal performance that sometimes outperforms conventional biological treatment systems [51]. Bharti and co-workers, for instance, reported that by optimizing light intensity, photoperiod and hydraulic retention time, total N and total P removal rates of microalgal systems could be as high as 70–95% and 60–90%, respectively [52]. Such removal values greatly exceed those usually realized by activated sludge systems or biofilm systems [53,54]. Importantly, nutrients captured by microalgae are retained in the harvested algae and not lost through volatilization or chemical precipitation; hence, they do not lose their potential to be utilized downstream [18]. From an engineering point of view, high photosynthetic fixation of carbon, efficient utilization of nutrients, and biomass accumulation consolidate the high potentiality of microalgae-based treatment systems as a standalone platform for nutrient recovery and the purification of wastewater (Figure 1).

This figure depicts how microalgal systems link together waste treatment and agriculture industries. The system makes use of wastewater from livestock farming, agricultural runoff and food processing industries. Pollutants are converted into productive biomass, thanks to photosynthesis and nutrient assimilation by microalgae. The end products (biofertilizers, animal feed, and biomass pellets) are incorporated in the agricultural value chain, thus delivering a closed nutrient cycle.

### 2.3. Microalgae as a System-Level Converter: From Pollutant Removal to Resource Recovery

Microalgal technology engineering importance goes beyond treatment efficiency and suggests a new approach on system design, moving from an end-of-pipe pollutant removal strategy to a resource-perspective approach. In microalgae-based systems, the nutrients commonly considered as wastes are intentionally retained and transformed into value-added biomass so that they can be recycled into agriculture-related supply chains [18,58]. Harvested microalgal biomass containing high N, P, and O atoms is enriched with organic carbon and can be used as bio-fertilizers, bio-stimulators, soil amendments [59], or as an input for downstream biorefinery processes [60,61]. Through such transitions, microalgal systems create a kind of connection between waste treatment and producing some agricultural input goods, so as to achieve a closed nutrient cycle at the system level. Nevertheless, the practical application of microalgae technology itself also depends on specific conditions. Previous studies show that this technology works best when dealing with wastewater with a relatively low or moderate degree of nutrients. At this time, there are usually many lighting conditions, and a complete resource recovery track has been established [62]. Further research proves that the three key parameters that limit the practical application of microalgae are light intensity, CO_2_ concentration and nutrient conditions [63]. Among these, ensuring that enough light is in the reactor and controlling the appropriate liquid depth are important factors in ensuring the activity of photosynthesis. As soon as light becomes insufficient, it will directly lead to a large drop in the absorption efficiency of nutrition and biomass, and it also makes it difficult for microalgae technology to independently treat high-nutrient wastewater [64]. Therefore, lack of light has become one of the main bottlenecks restricting the operational performance of microalgae systems. In practice, we can improve the efficiency of wastewater treatment by adding a source of artificial light and also reducing the liquid depth of the reactor. These biological and engineering sensitivities indicate that in many practical cases without proper operation conditions, microalgae alone might not be sufficient to meet the treatment objectives, particularly for high organic loading or complex wastewater, in which metabolic flexibility may be lost with photoautotrophic cultivation alone.

Based on this, when the organic load is high or the light intensity is low, the microalgae system usually has to be combined with other complementary treatment methods. For example, anaerobic digestion can be used prior to microalgae cultivation to lower the chemical oxygen demand (COD), or advanced pretreatment can be employed to enhance the wastewater’s quality [65]. Such treatments would stabilize the system’s operation and enhance the recuperation efficiency. A combination of microalgae cultivation and anaerobic digestion of high-level organic waste streams can improve the overall resource balance of the system. That is, organic matter is removed as biogas, and the resulting digestate is nutrient-enriched, available for the use of algae [66]. For this combined mode, the application of microalgae is not to directly take the place of the traditional wastewater treatment but rather to integrate the connection interface between environmental rescue and resource recovery (Figure 1). That is, by converting a waste substance into microalgae for bio-oil (microalgae biomass can be used as fodder), a microalgae-based system offers a feasible way to transform the use of agricultural waste disposal from merely fulfilling regulatory requirements to the comprehensive nutrient recovery of agricultural waste in a circular bioeconomy [67].

Although microalgae have certain potential as a nutrient recovery and a biomass-to-product conversion facility, their practical application cannot simply apply a single indicator such as nutrient recovery efficiency or biomass conversion efficiency. Instead, one has to think about the coordinated development of technology, economy and ecology in aggregate. On top of that, microalgae-related product quality needs to fit the standards and norms of specific industries. They need to be applied for functions such as improving the content of soil organic matter, soil water retention and soil microbial activity to really ensure that the comprehensive benefits of agricultural sustainable development can be achieved. Thus, a comprehensive consideration is needed, that is, it is needed to connect engineering feasibility and ecological functions in order to transform recovered nutrients into applicable products that can really realize circular agriculture well. Thus, here lies the use of biomass valorization as the link between process-level recovery of nutrients and system performance. Different valorization routes—spanning direct use as biofertilizers to the valorization in high-value biostimulant production—have different trade-offs with regard to energy use, costs and agricultural suitability. Hence, a deeper discussion of the applicable microalgal biomass valorization pathways is necessary to evaluate the practical prospects of microalgae-based circular agricultural systems. Following our discussion of the resource recovery pathways above, Section 3 introduces the downstream transformations and agricultural uses of microalgal biomass and stresses the impact product pathway choice has on the performance and scalability of overall system performance.

## 3. Biomass Valorization Pathways and Agricultural Applications

The effects of microalgae-mediated circular agriculture systems depend not only on the efficiency of upstream nutrient recovery, but more importantly on the downstream biomass valorization pathway and its capacity to decide the agricultural fate of the recovered resources. Unlike with other waste residues, microalgal biomass can act as a versatile carrier, the agronomic value of which is not intrinsic but is strongly driven by the valorization and conversion strategy chosen [67]. Through valorization pathways, microalgal biomass can be valorized in various value-added agricultural products, such as biofertilizers [68], biostimulants [69] and biopesticides [70]. Each valorization pathway puts different pressures on energy consumption and economics, and is differently integrated with current farming and agricultural practices and thus directly affects the sustainability and scalability of the system.

From an engineering and environmental perspective, biomass valorization must be regarded as a core system design element rather than a subsidiary end-of-pipe step. Life cycle assessments consistently identify biomass harvesting and drying as major contributors to energy demand and carbon emissions in microalgal systems, highlighting the need for low-impact processing strategies. The use of gravity sedimentation instead of chemical flocculation can substantially reduce environmental footprints while maintaining acceptable recovery efficiencies. Moreover, successful agricultural application of microalgal products requires addressing practical constraints related to nutrient bioavailability, product stability, and field performance [71]. Direct application of raw biomass may yield inconsistent agronomic outcomes due to slow nutrient mineralization, particularly for phosphorus; such limitations can be mitigated through conversion technologies, such as hydrothermal carbonation, which enhance nutrient availability and improve soil amendment properties [72]. Realizing the full potential of microalgae in closing an agricultural nutrient loop will ultimately require a holistic approach, which co-optimizes the valorization routes on the basis of profitability as well as agronomic performance, such that biomass conversion effectively benefits circular farms.

### 3.1. Biomass Valorization as a System-Level Design Decision

The effectiveness of microalgae-driven circular agriculture relies heavily on how the harvested biomass is valorized in agriculture [73]. Microalgal biomass is rich in proteins, polysaccharides, lipids, pigments and phytohormone-like substances and it is thus suited for a large number of downstream valorization applications such as biofertilizers, biostimulants, soil amendments and feedstocks for further transformation in biorefineries [74]. From a design perspective, the valorization of biomass is not a downstream step of use but a design aspect and core decision that also governs nutrient recycling efficacy, process energy demand, economic feasibility and environmental performance [75]. Different levels of processing lead to different functionalities: low-processing valorization pathways favour nutrient retention at a high scale, while higher-value valorization pathways favour controllable physiological regulatory effects at higher complexity and product processing [76]. The effectiveness of agriculture functionality can then be considered as the outcome of a valorization approach rather than as an artefact with a single product attribute.

### 3.2. Low-Processing Pathways: Nutrient Recycling and Soil Conditioning

Direct use of whole or minimally processed microalgal biomass is the lowest processing intensity and the most straight-forward application of circular agricultural principles. Harvested biomass—fresh slurry, dried biomass, or partly stabilized products—can be directly applied to soils as a source of nutrients and as an organic amendment [77]. A clear advantage of this approach is its slow nutrient-releasing dynamics. The nitrogen, phosphorus and potassium in microalgal biomass are mostly in organically bound forms and slow down release to soil thanks to microbial breakdown [78]. This is closely matched to crop nutrient demand and significantly reduces nutrient losses in terms of leaching and runoff with respect to customary mineral fertilizers, improving the nutrient-use efficiency at the field scale [79].

**Figure 2:**
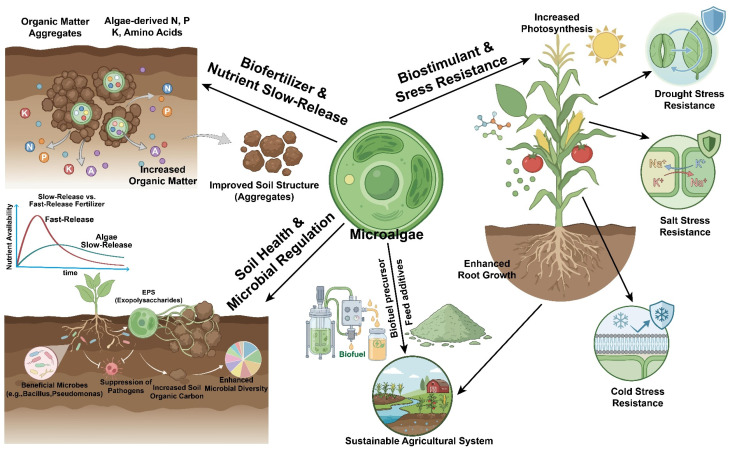
Schematic of the multifunctional mechanisms by which microalgae promote sustainable agriculture (created by the authors based on the literature [80,81,82,83,84]).

As discussed above, the incorporation of microalgal biomass improves soil physical properties and biological activities. More regular and repeated applications increase soil organic content, encourage soil aggregation, increase water-holding capacity and foster the microbial metabolism of soil—effects that are especially strong in degraded or organic-poor soils [80]. While microalgal biomass is relatively costly per unit volume and also variable in nutrients, it has low energy demand, high scalability and excellent agricultural compatibility, making it ideally suited for localized circular agricultural systems (Figure 2).

Figure 2 demonstrates how microalgae enhance agricultural systems through three major pathways: (1) Biofertilization and controlled nutrient release—By supplying nitrogen, phosphorus, potassium (N, P, K), and amino acids, promoting soil aggregate formation and enabling slow nutrient release (in contrast to quick-release fertilizers). (2) Soil health and microbial regulation—Secretion of extracellular polymeric substances (EPSs) stimulates the growth of beneficial microbes (e.g., Bacillus and Pseudomonas), increases microbial diversity, and suppresses pathogens. (3) Biostimulants and stress tolerance—Enhancement of plant photosynthesis and root development while improving resistance to abiotic stresses such as drought, salinity, and low temperature. (4) Bioenergy and feed applications—Microalgal biomass can be used as a biological precursor for producing clean bioenergy and feed additives. These applications provide renewable energy for agricultural activities and enhance livestock nutrition, thereby strengthening resource recycling and supporting a stable and sustainable agricultural system.

### 3.3. Moderate-Processing Pathways: Functional Stabilization and Agronomic Reliability

Processing strategies that fall into the range of those being moderately processed, such as drying, pelletizing, fermenting, co-composting, and formulation with other organic or mineral components, for instance, enhance the consistency, safety and agronomic reliability of microalgal products whilst keeping the energy and cost inputs acceptable [85]. This way of treating microalgal biomass transforms the microalgal biomass to a stabilized microalgal biofertilizer or amendment with more desirable properties for storage, handling and field performance [86]. At the level of moderately processed processing, microalgae are not only used as fertilizers. They can also be used efficiently for animal feed. For feed making, dried or slightly fermented microalgae (such as *Chlorella* and *Spirulina*) can be good feed additives. Dried microalgae (biofuel and feed are still under research and development) are rich in protein, polyunsaturated fatty acids and bioactive compounds. They are used for poultry, ruminants and aquaculture [87]. For instance, in new “land-free” circular farms, microalgae are cultivated from the livestock’s sewage. People first simply concentrate or dry them. Then, they can be directly used as liquid or pelletized feed for the animals [88]. Microalgae replace plant-based feeds. At the same time, they reduce the pressure on arable land. They make animal products richer. Also, they reduce the methane emissions by ruminant livestock [89]. Compared with direct application, moderately processed products have higher availability of bioactive substances and lower disease risk, while fermentation and co-composting lead to beneficial activity of microbes and accelerate the process of nutrient mineralisation [90], thus improving fertilizer-utilization efficiency and nutrient uptake in crops [91]. Additionally, in the conversion of bioenergy, microalgal biomass can also be turned into biofuels (biogas) by anaerobic digestion [92], which is also an important use. After this step, processing pathways are important intermediate pathways. In an agricultural circularity pathway, microalgae normally have a low carbon-to-nitrogen (C/N) ratio [93]. Thus, they are normally co-digested with carbon source materials (for example, crop straw, poultry manure or food waste) [94]. For example, microalgae can be fermented together with agricultural organic waste. In this pathway, renewable energy in the form of biogas can be produced, and digestate rich in nutrients can also be produced. The digestate can be reused to cultivate microalgae again. It can also be directly fertilized in farmland as liquid fertilizer [95]. In terms of system integration, such pathways balance performance improvement and engineering convenience, and integrate well with existing agricultural input supply chains. Although moderate processing unavoidably requires additional energy use and infrastructure, particularly in the drying and stabilization stages [96], its overall sustainability is greatly influenced by the local level of energy supply, the production scale and by the level of product homogeneity needed; still, in many circumstances in the field of agriculture, moderate-processing strategies represent a practical compromise between minimal energy cost nutrient recycling and product functionality.

### 3.4. High-Value Pathways: Biostimulants and Targeted Stress Regulation

Microalgae-driven circular agricultural biorefinery systems understand that HVPs are not just side-products but an independent subsystem affecting economic viability, market competitiveness and effectiveness of optimization schemes [97,98]. Biotechnology and systems engineering point out that optimization models are developed with only a focus on recovery efficiency and nutrient rationality, while, ignoring the valorization of HVPs, do not reflect the true technological competitiveness of the system and could result in unreliable AI models and multi-objective optimization for decision support. Thus, it is essential to explicitly include HVPs in overall process design and the expression of objective functions to promote realization of the concept of ‘‘no-waste operation” and industrial development of an integrated biorefinery [99]. HVPs harvested from microalgal biomass mainly include extracellular polysaccharides, free amino acids, carotenoids, antioxidant compounds and phytohormone-like bioactive compounds [100,101]. When applied to crops at low doses, these compounds can act as biostimulants to exert precise regulation of the signalling pathways for plant growth, improving plant resource use and stress adaptation [102,103,104]. When compared with a conventional fertilizer system that focuses on nutrient supply, biostimulants have the characteristics of high efficiency, selectivity and low input–high output to provide greater economic weight to HVPs in the entire system.

The stable production and directional synthesis of HVPs rely on engineering-level fine control of microalgal cultivation conditions [105]. Among them, light is not only a driving force for the growth of microalgal but also a metabolic switch that affects the distribution of carbon flux and induces secondary metabolite accumulation. Light intensity, spectral and photoperiod can be treated as designable, optimizable process variables that enable microalgae to synthesize different products such as proteins, polysaccharides, and antioxidant substances [64,106]. High light stress prompts *Haematococcus pluvialis* accumulates to accumulate astaxanthin for suppressing photo-oxidative harm [107], while for some kinds of microalgae, a particular light spectrum (for example, blue light) can induce the formation of bioactive polysaccharides [108]. In addition to light regulation, nutrient stresses such as nitrogen and phosphorus limitation and/or adjustments in the C/N ratio can reroute photosynthetic energy and carbon allocation towards the production of lipids, pigments and particular polysaccharides with a high added value [109]. These facts showcase that the formation of HVPs is not a passive phenomenon but a controlled metabolic result, an end product that can be achieved through smart process design. This controllability offers degrees of freedom that are very important to AI-supported dynamic optimization and control. Mindful of these strategic aspects of microalgal HVPs, we have to highlight that, beyond feedstocks, microalgal HVPs have very significant roles in the pharmaceutical and biomedical industries. In this respect, microalgae are increasingly considered irreplaceable natural biofactories [110] for conventional carotenoids but also for products with activity against viruses (for example, polysaccharides of *Spirulina*), against cancer (for example, astaxanthin) or beneficial for the nervous system [111,112]. These bioactive molecules typically have special structures and higher biological potency that are hard to obtain by traditional chemical synthesis and therefore have substantial commercial value and strategic significance in the development of drugs and functional food industries [113]. The combination of an agriculture-based microalgal cultivation system with a pharmaceutical-quality microalgal extract purification process could build a multi-track value-output biorefinery system, thereby closing value the loop from raw materials to final applications [114,115].

Overall, high-value pathways tightly knit together microalgal cultivation conditions, metabolic regulation and multi-tier application scopes, facilitating circular agriculture with economic and functional ends that go far beyond recovering a single nutrient objective. Explicitly incorporating HVPs into system designs and artificial intelligence (AI)-assisted optimization platforms bring them to be independent economic objectives or sub-objectives of system design, together with nutrient recovery efficiency, energy consumption and operating costs, inside a unified multi-objective function. Taking into account such integration helps to address the trade-off between resource circularity and value creation at the system level under dynamic and steady agricultural conditions, making decision-making fully reliable for scenario upgrades and the marketization of microalgae-based circular agriculture biorefinery systems.

### 3.5. Beyond Inputs: Soil Health Regulation and Microbiome Engineering

Apart from being nutrient carriers or biostimulants, microalgae are increasingly functioning as soil regulators and driver agents of soil health and microbial ecology [116]. Microalgae photosynthetically fix CO_2_ and secrete extracellular polysaccharides, proteins and various metabolites for soil organic carbon, directly contributing to the soil organic carbon pool and causing the formation of stable soil aggregates [117], thereby improving soil health, water-holding capacity and the long-term potential for soil carbon sequestration [59] (Figure 2). Meanwhile, microalgae also strongly regulate the soil microbial community structure and activity; their remains and extracellular substances can supply ready-made carbon and energy for promoting the activity of soil microbes, enriching community diversity and facilitating beneficial functional groups [118,119]. Sequestrate synergistic interactions with plant growth-promoting microorganisms improve the efficiency of nutrient mobilization and facilitate the inhibition of soil-borne pathogens via competition and the accumulation of antimicrobial products [120]. In contaminated or deteriorated soils, microalgae also participate in soil remediation by promoting nutrient activation, biological nitrogen fixation [121], immobilization of heavy metals and the improvement of soil under saline–alkali conditions [122]. Thus, by collectively remodelling soil physicochemical properties and microbial communities, microalgae help build up robust “microalgae–microbe–plant” interaction frameworks that can promote high-efficiency agricultural production and meet the goal of decreasing chemicals consumption.

### 3.6. System-Level Trade-Offs and Pathway Selection

At the system level, microalgal biomass valorization pathways cover a full spectrum with a continuously increasing processing intensity, energy input and function specificity. Low-processing valorization routes focus on the scalability, robustness and maximal protection of nutrients; high-value valorization pathways focus on targeted physiological control and higher value at the expense of higher technical complexity and energy inputs; and intermediate valorization routes can provide intermediate compromises between these two extremes. Crucially, none of the valorization pathways is the overall optimum; the correct choice of valorization is strongly context dependent and will take into account site-specific factors such as waste characteristics, local energy resource, agricultural market needs, infrastructural challenges and overall management goals. As such, biomass valorization needs to be seen as an integral part of system design and not merely as a downstream consideration, as it plays the fundamental role in nutrient cycle efficiency, energy balance and system performance. Thus, this system-level perspective is a critical starting point to form integrated deployment strategies and later to optimize them.

## 4. System Integration Pathways of Microalgae in Sustainable Agriculture and the Circular Economy

### 4.1. Microalgae-Driven Agricultural Circular System Models

**Figure 3:**
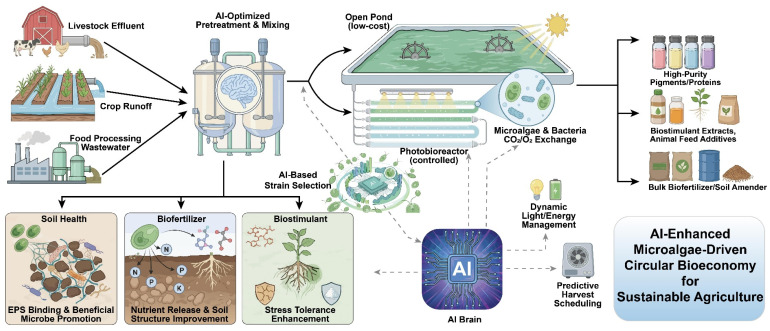
Schematic of an AI-enabled, microalgae-driven sustainable agricultural circular bioeconomy (created by the authors based on the literature [123,124,125,126,127,128]).

For an engineering system design of microalgae applications for agricultural circular systems, there is no single integrable option or best industrial configuration; instead, reasonable integrations should be determined by multidimensional trade-offs [123]. Precisely speaking, system design needs to simultaneously consider the stability of resource supply, input energy consumption, running costs, the value structure of the output, and the spatial–temporal matching of agricultural production processes. According to this, microalgae systems can be flexibly embedded in different kinds of agricultural circular system modes, and different integration pathways leading towards nutrient loops or value upgrading will emerge (Figure 3).

AI-assisted genomic screening is first used to find good microalgal strains for wastewater treatment and microalgal biomass. The AI system uses organic wastewater from livestock, farmland and the food processing industry as the input, which is then fed to the cultivation system (open ponds or photobioreactors) after AI optimization pretreatment and blending. The ‘AI brain’ (such as light control and harvesting time prediction) realizes real-time management; then, microalgae grow effectively and are converted into high-purity pigments, feed additive and biofertilizer, which can be used to improve the condition of fields, supply bio-nutrients and improve crop tolerance, closing a loop of recycling wastewater for the sustainable development of agriculture.

Within circular agriculture, given their high assimilation and bioconversion efficiencies, microalgae function as an interface for linking waste valorization with green agriculture production [129,130]. Starting from the viewpoint of system integration, microalgae are generally integrated into circular agricultural systems in the following roles: (1) as wastewater treatment and recovery units to remediate nutrient-containing wastewaters; (2) as bioconversion systems that convert carbon, nitrogen and phosphorus resources from waste into valuable biomass; and (3) as the heart of a multiproduct biorefinery system that simultaneously delivers valuable products such as biostimulants and feed additives. Depending on the role of a given system and system boundaries, two main integration schemes are proposed.

Another type is the simple circulation model for “nutrient loop closure”, such as the typical circulation pathway: “livestock and poultry wastewater–microalgal cultivation–algal fertilizer application to fields”. This model focuses more on direct recycling of agricultural nutrients, for which the livestock wastewater is used as the growth medium of microalgae, and then the microalgae is extracted to form the algal slurry through a very simple production process, which is applied directly as liquid fertilizer or liquid soil conditioner [131]. Due to a relatively simple process and high energy efficiency, this model is suitable for moderately sized livestock operations with nearby arable land to assimilate nutrients, thereby realizing in situ and low-cost cycle reuse of core nutrients such as nitrogen and phosphorus.

This results in a “product-oriented” integrated value-addition model, which forms a chain of “diverse agricultural wastewaters ⟶ microalgal growth ⟶ multiple-path product development”. Microalgal technologies provide specific pathways in circular agriculture, ranging from practical circular loops of agricultural nutrients to relatively advanced circular industry integration. At their most simple level, microalgae serve agricultural pollution mitigation. A more technologically integrated model broadens the roles of microalgae: in addition to bioremediation, microalgal biomass is used for marketable products such as high-purity extracts, biofuels and specialty feed ingredients. This scheme improves economic sustainability by offsetting the cost of operation [132]; meanwhile, it increases process technology complexity and the dependence on the market, which is more appropriate for large-scale biorefineries or local treatment facilities. The selection of these two pathways comes ultimately from different environmental objectives, different economic targets and regional characteristics.

### 4.2. Engineering Scale-Up and Economic Feasibility Challenges

Although microalgae have great application potential and some remarkable environmental advantages at the laboratory and small-experimental scales [133], the application of microalgae at the large-scale level in agricultural circular systems still has plenty of technical and economic issues [134]. Differing from linear technology pathways, the scale-up process of microalgae cultivation technologies cannot be reduced to a simple technique geometry scale-up but should be treated as an ongoing closed feedback and loop process of “scale-up–evaluate feedback–reoptimize” [135]. In an open and dynamic agricultural environment, several technical and economic feasibility problems still exist [136].

Cultivation systems and energy efficiency are the two main bottlenecks of large-scale production. The photoautotrophic growth of microalgae is heavily dependent on light and the adopted illumination strategy directly determines the cost and sustainability of the whole system [108]. Open raceway ponds have a simple structure and low investment costs and a basis for application in scenarios where the land is abundant [137]. However, open raceway ponds have their own inherent limitations: low light utilization efficiency, a high evaporation rate and a high possibility of contamination; thus, they face huge obstacles when operating in high-latitude regions or in winter (depending on natural light would increase the operational energy costs enormously) [138,139]. Contrary to the open photobioreactors mentioned above, closed photobioreactors have the advantages of controllability, high cultivation density and pollution control [136]. Studies have demonstrated that flat-panel, tubular, thin-film photobioreactors can largely improve light utilization efficiency and cell density [140], which makes the cultivation process more controllable and the achieved high cell density lowers the volumetric load and energy consumed in the following harvesting step (30–20% of the production cost in general [141]). Although investment in closed photobioreactors is high in advance, life cycle assessment and techno-economic analysis show that, for a special agricultural case, the yield advantage can be translated to economically favourable performance [142]. For example, when treating meat processing wastewater, a closed photobioreactor achieved a biomass productivity of 483.33 mg·L^−1^·d^−1^, which is more than five times higher than that of an open-pond system (95.00 mg·L^−1^·d^−1^), and also achieved 100% ammonium nitrogen removal; it also greatly decreased the energy used in the harvesting process and land footprint per unit biomass productivity in the closed photobioreactor [143]. Therefore, when facing land shortage and/or for HVP production, closed photobioreactors might be better than the usual open-pond system [144].

System design and operation have to be extremely flexible for the intermittent and seasonal discharge of agricultural waste [25]. The generation of livestock wastewater or processing by-products can dramatically change with agricultural production cycles [145], and is incompatible with stable and effective growth of microalgae. For microalgae engineering, the traditional “fixed-conditions” concept should be abandoned for a scale-up concept towards a dynamic synergy between processing capacity and the variable load on the system’s input. It requires an engineering system on a modular, “core-variable” basis, complemented by proper buffering and regulating units. The core of this engineering approach is a rigorous and closed-loop approach: reliable scale-up is not a straight, ever-forward process, but a cycle of “scale-up and scale-down” [146]. In particular, problems such as poor mixing, mass transfer limitations or physiological problems met during the pilot plant scale-up should also be treated as a signal that recommends subsequent “scale-down” design investigations in the lab. By going back to the lab to investigate mechanisms and tune up parameters [147,148], and then returning to the next design iteration to give back learned scientific insights, system stability and system efficiency are gradually improved. Such iteration cycles lead to scientific and reliable engineering scale-up. Through this cyclic iteration, the risk of engineering scaling-up failure can be decreased and this is the very important and core method for linking basic research with commercial application and fulfilling the engineering scaling-up process. With respect to economic feasibility, the economic competitiveness of a microalgae system does not rely on one factor only, but has multiple important factors such as energy efficiency, capital investment (CAPEX), operating cost (OPEX), stability of the system and flow of by-products and HVPs [149,150]. Various technological and economic assessments indicate huge differences for the economic performances of different cultivation systems and large engineering scale-up can further contribute to reducing unit production costs [151].

For large-scale systems, the design and operation optimization should be based on the technical and economic objective function, rather than considering solely the biological production rate or nutrient removal efficiency. Based on the construction of a target function from different characters such as energy consumption, investment and product value, different system structures, system operating modes and product paths can be quantified in a unified multi-objective trade-off space. This has set up a clear and transparent decision-making system for next-step AI-based modelling and optimization, enabling data-based trade-offs between resource recovery efficiency and economic performance.

### 4.3. Techno-Economic Feasibility and Multi-Product Synergy Strategies

In agriculture, microalgal systems designed for a single low-value product, such as normal biofertilizers, tend to be non-viable, as product revenues do not cover the cost of microalgal cultivation, harvesting and processing. Bypassing this bottleneck requires shifting to a multi-product, biorefinery approach in which microalgal biomass is valorized in successive uses and by product value extraction to get the maximum value of the system and spread the cost.

Specifically, the above strategy is for product portfolio diversity. Most of the microalgal biomass can be processed cost-effectively—for example, slurry preparation or simple drying—and then directly used as high-quality organic fertilizer or soil conditioner, achieving its main nutrition recycling role and bringing the basic revenue to a stable level [152]. At the same time, part of the microalgal biomass is processed to extract high-value products, such as certain polysaccharides, proteins or pigments, which can be developed to different biostimulants, feed additives, natural pigments, etc. [153]. Such a “baseline primary product with value-added co-products” development model internalizes the cost of agricultural waste treatment for the environment, but shifts the burden to high-value and marketable products, greatly enhancing the stability to the project’s business operation and overall robustness (Figure 3).

Consequently, future engineering design of microalgae-based agriculture systems has to achieve a fundamental change in paradigm: going from improving the performance of isolated micro-algal components, such as that of the photobioreactor efficiency, towards integrated system designs with a target at net benefits of the whole system. Implementing this from scratch means that feedstocks, pathways, energy networks, products, and environmental aspects are all simultaneously considered. Multi-objective optimization finds the optimal trade-off between technical and economic feasibility. The adoption of a system-integrated, multi-product biorefinery approach is essential to scale microalgal technologies from pilots to full agricultural applications and to achieve efficient integration of such technologies into a circular economy, as proposed recently [113].

### 4.4. Artificial Intelligence-Assisted Optimization of Microalgae-Based Agricultural Systems

The increasing complexity of microalgae-driven circular agriculture, as reflected in the strong spatiotemporal dependence of feedstock properties, of growing conditions and of system performance, reveals the limits of straightforward empirical optimization [154]. New research indicates that AI- and data-driven methods can help most in combination with mechanistic knowledge and be implemented within a well-defined framework of control structures instead of as so-called point solutions [155,156]. Their effectiveness is ultimately a function of good data and the clear understanding of the biological systems involved in circular agriculture. In this case, AI mostly plays mostly the role of a tool that enhances instead of replaces the existing scientific and engineering knowledge. A full AI-supported approach therefore needs to start with biological mechanisms followed by process control and system-level integration.

Within microalgae-based agricultural cycles, the effective use of AI begins with the identification and design of engineered strains that match specific application goals. Methods of strain screening can be slow, labour-intensive and uncertain [157]. The rapid development of AI has started to tackle this upstream challenge [158]. Machine learning models can incorporate information from multi-omics data, including from genomics [159], transcriptomics and metabolomics, to identify genetic markers associated with efficient nutrient uptake, strong environmental tolerance and the synthesis of high-value compounds [160]. By combining physiological data for algal strains with simulations of different environmental scenarios, AI models can also predict phenotypic performance for different complex wastewater substrates and fluctuating climatic scenarios. These predictions can orientate high-throughput phenotypic screening, increase the efficiency of screening and shorten the development time [161]. This step supplies the biological basis for subsequent process optimization and process integration [162]. In practice, AI has mostly played the role of an accelerator in this stage, and its predictions still need experimental identification, verification, and detailed mechanistic analysis.

Once high-performance engineered strains have been obtained, fine control of cultivation conditions is key to improving the overall performance of the system. Microalgal growth occurs with variation in several parameters simultaneously, such as nutrients, light and temperature. These variables, and how they interact, in complex and nonlinear ways, often make it challenging for simple mechanistic models, based on various assumptions and simplifications, to offer a precise description. AI offers practical ways to resolve this challenge. Some data-driven methods are also available (including ML [163], artificial neural networks [164], support vector regression [165], random forests and long short-term memory networks (LSTMs) [166]) to learn patterns and correlations from historical and real-time data. They are able to describe the system dynamics and predict key parameters such as biomass productivity and nutrient removal efficiency [167]. Based on the predictions, AI models can even aid in dynamic planning and real-time tuning of operating parameters [168]. Therefore, the parameters of hydraulic retention time, nutrient load, light regulation measures and harvest duration, among others, can be adjusted all the time so as to keep high levels of nutrient recoveries and biomass production under variabilities [169]. These RL methods are natural for this problem because they constantly update operation strategies with feedback, which lets cultivation systems move from fixed conditions to adaptive control. These AI models can also be blended with optimization methods, such as Bayesian optimization and genetic algorithms, to enhance operation strategies and to reconcile different objectives (for example, productivity, energy and resource use) [57].

At the system level, microalgae technologies need to be integrated with different types of farms, agricultural land (crop land), and energy infrastructures. Such integration also leads to complex problems of decision-making that involve competing objectives, long-term uncertainty and matching of multiple dynamic flows [170]. AI-assisted decision-support frameworks are helpful in such contexts. Combining AI-based surrogate models with multi-objective optimization algorithms such as genetic algorithms and particle swarm optimization, researchers can compare various integration schemes. Examples include coupling microalgae cultivation with aquaculture treatment waters or using microalgae for greenhouse CO_2_ consumption. These approaches enable quantitative evaluations of the use of energy, economic return and environmental impact, which can help to identify the appropriate system configurations for the application [128,171]. Agricultural wastes also vary in both their flow and composition [172]. AI-based predictive models can aggregate historical records with climate information to estimate the future load of a particular nutrient. Such predictions can help operational scheduling and adaptive management in advance to ensure the stable performance of nutrient treatment [173]. AI can also link different analytical approaches. Process models, LCA, and techno-economic analysis (TEA) can be brought together on a common data structure, and can also be evaluated at the process, system and sustainability levels. To achieve economically viable microalgae-based circular agriculture, AI-assisted optimization must be based on a well-defined techno-economic objective function (OF). In the same way as with the biorefinery concept, the aim is not only maximizing microalgae biomass production. The economic outcome is driven by producing HVPs, such as drugs and bioactive compounds [174,175]. Therefore, a reliable optimization framework should simultaneously factor in: the genetic potential of targeted strains; dynamic environmental regulation strategies such as light and nutrient stress; and overall techno-economic feasibility. Mathematically speaking, the AI algorithm should maximize total profit *Z* over the length of cultivation time period *T* based on a biorefinery framework optimization model [176].

$$Maximize\;Z=\int_{0}^{T}\left[\sum_{i}P_{HVP,i}\cdot Y_{HVP,i}\left(S,U(t))\right.+P_{bulk}\cdot Y_{bulk}\left(S,U(t))\right.-C\left(U\left(t\right)\right)\right]dt-CAPEX$$

In this objective function, the variables are defined as follows:

*S* (strain selection) represents the initial boundary condition of the system. Selecting a suitable microalgae strain determines the theoretical upper limit for producing high-value products [177]. Without high-performance strains, improvements in later process stages remain limited.

*U*(*t*) (dynamic operating conditions) represents the control variables in the optimization process, mainly including real-time regulation of light strategies and nutrient stress. By adjusting *U*(*t*) through AI-supported control, targeted stress conditions can be applied to redirect cellular metabolism toward the accumulation of high-value products such as specific bioactive compounds [178].

*Y* and *P* denote product output and market price. *Y_HVP,i_* represents the dynamic yield of the i-th high-value product and corresponds to its market price *P_HVP,i_*. This component is the main contributor to economic performance because the revenue from HVPs is typically much higher than that from bulk biomass products.

*C*(*U*(*t*)) and *CAPEX* represent operating costs and capital expenditure, including the cost of maintaining facilities such as closed photobioreactors [179].

By using this techno-economic objective function, the dynamic programming algorithm is able to assess the trade-off between the high cost of closed photobioreactors and the returns from high-value products. This function assists in the rapid, efficient, low-waste transition from laboratory to industrial-scale production and ensures that this technical approach remains economically competitive. The bottom line is that AI provides a systematic way to optimize microalgae-driven circular agriculture in a hierarchic structure of “algae species design–process regulation–system integration design”. However, it must be clear that within this paradigm, the core role of artificial intelligence is as an augmentative tool that empowers and improves the science foundations and engineering of the system rather than a system that solves problems autonomously.

### 4.5. Performance-Based Comparison and System-Level Evaluation

From the system-level perspective, microalgae-based platforms should be evaluated holistically using integrated system-level metrics, such as nutrient recovery efficiency, energy demand, greenhouse gas mitigation and their impact on farming practices. As summarized in Table 1, microalgae-based systems usually have higher nitrogen and phosphorus recovery efficiencies than existing technologies such as composting, anaerobic digestion, constructed wetlands or chemical precipitation; meanwhile, they generate multifunctional biomass suitable for direct recycling into agriculture. The system includes multifunctionality—from biofertilizers and biostimulants to soil amendments—and has a specific advantage for circular agricultural uses, because it couples waste treatment with the recycling of nutrients and product substitution. However, Table 1 also shows that this benefit has a moderate-to-high energy cost (on harvesting, dewatering and downstream processing) that might offset environmental benefits if not managed. By contrast, conventional technologies are lower in energy requirements or higher in robustness of operation, but often present with poorer nutrient exploitation, product capability or potential for deployment and integration. Consequently, the overall success of microalgal systems does not require maximum realization of any single performance index, but, instead, requires trade-offs to be balanced between nutrient harvest, energy input and product use in any given situation. This comparison illustrates the value of integrated system-level design and pathway choice, rather than the isolation of process optimizations, when assessing the role of microalgae in circular agricultural systems.

## 5. Challenges and Future Perspectives

Even though the multifunctional potentials of microalgae in agricultural circular systems have already been widely verified, the large-scale use of microalgae still faces systematic challenges [187]. Among them, strain selection, as the first step of the microalgae technology path, is the key foundation for guaranteeing the stability of the system, the yield and its economy. However, at present, existing studies are mostly committed to engineering scaling-up and process optimization but pay no attention to the systematic methodology of strain screening [135,188]. For future studies, a goal-oriented strain screening system for particular agricultural scenarios should be generated, taking into account various factors, including growth rate, efficiency of the utilization of light energy, absorption capacities of nutrients and even pollution resistance and stress resistance, as well as the synthetic potential of HVPs [189]. Using high-throughput screening methods, metabolomics analysis and genomic-assisted selection, a functional strain library suited to different types of wastewaters and climate conditions can be constructed, to provide a biological basis to inform subsequent process optimization and large-scale scaling-up.

At the engineering level, scaling up microalgae circular agriculture involves not only expansion of the equipment and infrastructure but also a systems engineering process that synergistically balances the biological limit, process design and economics objectives [190]. Future research should strengthen the coupled design between “strain–process–system”, achieving a dynamic balance between nutrient recovery efficiency and energy consumption control by improving light energy utilization efficiency, optimizing reactor structure, and constructing stable algae–microbe symbiotic systems [191]. At the same time, engineering scale-up should also follow a closed-loop approach of “scale-up–scale-down” bidirectional confirmation to guarantee the transferable and stable factors of parameters in various scales. At the level of system integration, deep integration of microalgae technology into existing agriculture infrastructures represents a path towards increasing its techno-economic feasibility. For example, when embedding microalgae cultivation into livestock and poultry farming systems [192], facility agriculture or agricultural wastewater treatment plants can accomplish on-site nutrient recycling and effective use of spatial utilization [193]. Constructing modular models for recycling according to regional resource endowments can increase the adaptability of the recycling system to fluctuations in raw materials and environmental changes (Table 2).

Another important direction for improving the benefit of the system as a whole is to diversify product utilization pathways. In addition to being biofertilizers and biostimulants, microalgae biomass can also be high-tech transformed into feed additives, functional materials and active substances for pharmaceuticals [199]. Future research efforts need to shift from a single-product development for microalgae to a multi-product cooperative development that brings about nutrient recovery, biomass production, high-value-added compounds that are complementary with each other and compose a multi-product value stream [200]. Meanwhile, this would also enhance the economic sustainability of the system. By taking advantage of digital and smart technologies, new opportunities in microalgae circular agriculture can also be created. The integration of the sensor network, automatic control and AI-based statistics can deliver real-time control and dynamic optimization of the process [201]. Thereafter, AI-based LCA and TEA can also assist scenario assessment for different strains, system designs and product selections. These tools assess the main trade-offs—such as between energy input, carbon mitigation and economic return—to provide a scientific basis for technology deployment strategies.

Overall, the future development of microalgae circular agriculture systems should shift from single-technology optimization to a comprehensive framework that synergistically promotes strain selection, engineering design, system integration, and multi-objective optimization. Only by establishing a close coupling between biological fundamentals, engineering methods, and digital optimization can microalgae technology be transformed from laboratory innovation to large-scale, economically feasible, and environmentally sustainable agricultural applications.

## 6. Conclusions

Microalgae-driven circular agriculture is an important pathway for the resource utilization of agricultural waste, closed-loop recycling of nutrients from agriculture and a low-carbon transformation of agriculture. Basically, in this review, we summarize the major functions of microalgae in nutrient recovery, microalgae biomass valorization and system integration. It also highlights that large-scale and sustainable development must rely on a multi-level collaborative optimization development process to optimize the strain, process and system. Within this system, strain screening and evaluation of the genetic potential are the biological basis of the technological system: both influence the efficiency of nutrient conversion and the generation of high-value products. Engineering-scale expansion should be checked with a closed-loop approach that integrates scale-up and scale-down. Such a strategy will guarantee the stability and transferability of parameters for different operation scales and boost the coupling between microalgae units and the agriculture structure. At the level of the economic structure, coordinated development of HVPs is important for HVPs to improve economic feasibility and increase the capability of the system to develop an economic risk buffer. It should be included into a unified multi-objective techno-economic function alongside nutrient recovery efficiency and the energy utilization level. Artificial intelligence and digital modelling are key tools for the dynamic optimization of complex systems. However, their practical application requires the construction of an objective function proposed from the scientific perspective and the build-up of complete system components. To summarize, a microalgae-driven circular agriculture is a multi-variable, multi-constraint, and multi-objective system optimization problem. Long-term sustainability requires a coordinated balance of biological performance, engineering parameters and economic structure. Future research should strengthen the coupling between strain design and system-level optimization, build transparent AI-assisted decision-making models and examine techno-economic and ecological performance, to be realized under real agricultural conditions. Such work would facilitate the large-scale employment of microalgae technology and facilitate the transformation and upgrading of the circular agricultural system.

## Figures and Tables

**Table 1:** Integrated comparison of microalgae-based systems and conventional technologies for nutrient recovery and circular agricultural applications.

Technology	Target Waste Stream	Primary Function	Nutrient Recovery	Energy Demand	Product Form and Value	Agricultural Compatibility	Limitations	System Integration Potential	Refs.
Microalgae-based systems	Agricultural/livestock wastewater	Nutrient recovery/biomass production	High	Low–moderate	Multifunctional biomass (biofertilizers/biostimulants/soil amendments)	High (direct land return, slow-release nutrients, bioactive effects)	Harvesting cost; light and temperature dependency; wastewater variability	High (couples waste treatment, nutrient cycling, and agricultural inputs)	[180]
Activated sludge	Agricultural wastewater	Biological wastewater treatment	Moderate	High	Low-value sludge	Low	High energy consumption; sludge disposal	Low	[181]
Anaerobic digestion	Livestock manure	Organic matter stabilization/biogas	Moderate	Moderate	Biogas/digestate	Moderate (digestate nutrient imbalance)	Digestate management; limited nutrient valorization	Moderate	[182]
Composting	Organic agricultural waste	Organic waste stabilization	Low–moderate	Low	Compost	High (widely accepted soil amendment)	Long processing time; N volatilization	Low	[183]
Constructed wetlands	Agricultural runoff	Wastewater treatment	Moderate	Low	Plant biomass with limited reuse	Low–moderate	Large land requirement; seasonal variability	Low	[184]
Chemical precipitation	Wastewater (P-rich streams)	Targeted phosphorus removal	High	Low–moderate	Mineral precipitates	Moderate (requires post-processing)	Chemical input dependence	Low	[185]
Membrane separation	Concentrated wastewater	Nutrient concentration/separation	High	High	Concentrated nutrient streams	Low (indirect agricultural use)	Fouling; high operational cost	Low	[186]

**Table 2:** Comparison of microalgae integration scenarios in circular agricultural systems.

Integration Scenario	Waste/Resource Inputs	Core System Function	Nutrient Recovery Performance	Energy Demand	Biomass Utilization Pathway	Agricultural Compatibility	Engineering Challenges	Refs.
Livestock wastewater–microalgae–cropland system	Livestock and poultry wastewater	Wastewater treatment/nutrient regeneration	High	Moderate	Biofertilizer/soil amendment	High	Variability of effluent composition; pathogen control	[194]
Anaerobic digestion–microalgae coupled system	Digestate and CO_2_-rich biogas	Residual nutrient recovery/carbon capture	Moderate	Moderate-high	Biomass valorization/energy offset	Moderate	Energy trade-offs; system complexity	[195]
Agricultural runoff–open-pond microalgae system	Diffuse nutrient runoff	Nutrient interception and polishing	Moderate	Low	Low-processed biomass/recycling	Moderate	Seasonal instability; land requirement	[196]
Photobioreactor-based microalgae treatment	Controlled wastewater streams	High-efficiency nutrient recovery	High	High	High-value biomass/extracts	Moderate	Capital and energy intensity	[197]
Integrated microalgae–biorefinery system	Mixed agricultural wastes	Nutrient recovery + multi-product valorization	High	Very high	Biostimulants, specialty products	Low to moderate	Economic feasibility; process coupling	[198]

## Data Availability

No new data were created or analyzed in this study.
